# High-resolution compressed sensing time-of-flight MR angiography outperforms CT angiography for evaluating patients with Moyamoya disease after surgical revascularization

**DOI:** 10.1186/s12880-022-00790-w

**Published:** 2022-04-07

**Authors:** Shujing Ren, Wei Wu, Chunqiu Su, Qianmiao Zhu, Michaela Schmidt, Yi Sun, Christoph Forman, Peter Speier, Xunning Hong, Shanshan Lu

**Affiliations:** 1 Department of Radiology, The First Affiliated Hospital of Nanjing Medical University, Nanjing, Jiangsu Province China; 2 Department of Neurosurgery, The First Affiliated Hospital of Nanjing Medical University, Nanjing, Jiangsu Province China; 3 Siemens Healthcare GmbH, Erlangen, Germany; 4 MR Collaboration NE Asia, Siemens Healthcare, Shanghai, China

**Keywords:** Magnetic resonance angiography, Compressed sensing, Moyamoya disease, Cerebral revascularization

## Abstract

**Background:**

To evaluate the utility of high-resolution compressed sensing time-of-fight MR angiography (CS TOF-MRA) for assessing patients with moyamoya disease (MMD) after surgical revascularization, by comparison with computer tomography angiography (CTA).

**Methods:**

Twenty patients with MMD after surgical revascularizations who underwent CS TOF-MRA and CTA were collected. The scan time of CS TOF-MRA was 5 min and 4 s, with a reconstructed resolution of 0.4 × 0.4 × 0.4 mm^3^. Visualization of superficial temporal artery and middle cerebral artery (STA–MCA) bypass, neovascularization into the brain pial surface and Moyamoya vessels (MMVs) were independently ranked by two neuroradiologists on CS TOF-MRA and CTA, respectively. The patency of anastomosis was assessed as patent or occluded, using digital subtraction angiography and expert’s consensus as ground truth. Interobserver agreement was calculated using the weighted kappa statistic. Wilcoxon signed-rank or Chi-square test was performed to investigate diagnostic difference between CS TOF-MRA and CTA.

**Results:**

Twenty-two hemispheres from 20 patients were analyzed. The inter-reader agreement for evaluating STA–MCA bypass, neovascularization and anastomosis patency was good to excellent (*κ*_CS TOF-MRA_, 0.738–1.000; *κ*_CTA_, 0.743–0.909). The STA–MCA bypass and MMVs were better visualized on CS TOF-MRA than CTA (both *P* < 0.05). CS TOF-MRA had a higher sensitivity than CTA (94.7% vs. 73.7%) for visualizing anastomoses. Neovascularization was better observed in 13 (59.1%) sides on CS TOF-MRA, in comparison to 7 (31.8%) sides on CTA images (*P* = 0.005).

**Conclusion:**

High-resolution CS TOF-MRA outperforms CTA for visualization of STA–MCA bypass, neovascularization and MMVs within a clinically reasonable time in MMD patients after revascularization.

## Background

Moyamoya disease (MMD) is a progressive steno-occlusive disease which leads to an abnormal vascular network at the base of the brain [1]. Direct revascularization such as anastomosis of the superficial temporal artery (STA) and middle cerebral artery (MCA) combined with indirect revascularization such as encephalo-duroarterio-myo-synangiosis (EDAMS) is an effective procedure for MMD patients with ischemic symptoms [2, 3]. Despite initially successful surgical procedure which has a high anastomosis patency rate of 88–97% in MMD [4, 5], the risk of bypass occlusion may lead to recurrent ischemic events and deteriorated neurological outcome [6, 7]. Moreover, visualization of neovascularization into the brain pial surface after indirect bypass and the reduction of burden on fragile Moyamoya vessels (MMVs) would also be helpful for surgeons to evaluate the effectiveness of this procedure [8]. Thus, long-term follow up after surgical revascularization is extremely important for MMD patients.

Digital subtraction angiography (DSA) has long been the gold standard for assessing the patency of the STA–MCA anastomosis postoperatively, but it is an invasive, radiation-associated technique with the possibility of complications. Computer tomography angiography (CTA) is the most commonly used technique to evaluate and follow up patients with STA–MCA bypass [9, 10]. However, CTA suffers from several disadvantages including the need for contrast medium, radiation exposure and the interference from the skull. Previously, noninvasive and radiation-free techniques, such as time-of-flight MR angiography (TOF-MRA), has also been used to evaluate bypass patency after cerebral revascularization surgery [11, 12], which can be repeatedly used for follow-up examinations and especially benefit for pediatric patients. However, it has limitations in the visualization of small and distal arterial branches. Moreover, the resolution is often limited due to the long scan time which is not acceptable in a real clinical setting. Higher image resolution with shorter scan time is an urgent requirement.

Recently, the application of compressed sensing (CS) theory in MRI has aroused much interest among clinical researchers. CS is based on the principle that unaliased images can be reconstructed from a reduced number of k-space samples by exploiting image compressibility or sparsity [13]. Compared with conventional approaches, CS allows rapid acquisition of images by means of k-space undersampling [14]. This is clinically significant because motion artifacts can be decreased in a shortened scan time. Our two previous studies found that CS TOF-MRA could provide adequate image quality for the diagnosis of head and neck arterial steno-occlusive disease within a reasonable acquisition time [15, 16]. Furthermore, CS TOF-MRA is benefit for displaying small and distal vessels [17–19]. To the best of our knowledge, the diagnostic performance of CS TOF-MRA for assessment of revascularization in MMD patients has not yet been evaluated.

In this study, we aimed to investigate the clinical utility of CS TOF-MRA for assessment of STA–MCA bypass, neovascularization and MMVs after surgical revascularization in MMD patients, by comparison with traditional CTA technique.

## Materials and methods

### Patient selection

This prospective study was reviewed and approved by the local Institutional Review Board. Informed consent was obtained from all the patients. From July 2019 to July 2021, CS TOF-MRA and CTA were performed in 22 MMD patients for evaluation after surgical revascularization. The detailed inclusion criteria were as follows: (1) time interval between CS TOF-MRA and CTA was less than 2 weeks; (2) good image quality without artifacts; (3) complete clinical data could be acquired.

### Compressed sensing TOF-MRA protocols

CS TOF-MRA was performed based on a research sequence on a 3.0 T MR scanner (MAGNETOM Skyra, Siemens Healthcare, Erlangen, Germany) by using a 20-channel head/neck coil. The parameters were as follows: TR 21 ms, TE 3.49 ms, flip angle 18°, FOV 220 × 200 mm^2^ (the scan range extended from C5 of the ICA to M4 of the MCA, covering both the STA–MCA bypass and the circle of Willis), matrix 368 × 334, slice thickness 0.4 mm, and number of slabs 4. The acquired voxel size was 0.6 × 0.6 × 0.6 mm^3^ and reconstructed to 0.4 × 0.4 × 0.4 mm^3^. The acceleration factor of CS TOF-MRA was set at 5, without using phase or slice partial Fourier. The total acquisition time was 5 min and 4 s. Data were reconstructed using 10 iterations of the modified fast iterative shrinkage-thresholding algorithm. The reconstruction time was 1 min and 18 s. Maximum intensity projection (MIP) images were reconstructed in axial, coronal and sagittal views.

### CT Angiography protocols

CTA examinations were performed in all MMD patients after surgical revascularization using a 256-row MDCT scanner (Revolution, GE Healthcare, Waukesha, WI, United States). The following 3-dimensional CTA scanning parameters were used: collimation, 0.625 mm × 32, 25-cm field of view, tube voltage, 120 kV, 320-mA tube current, matrix 512 × 512, 0.28 s per gantry rotation, and pitch of 0.992. The raw data was reconstructed with 0.625-mm slice thickness and 0.625-mm slice interval. The voxel size was 0.488 × 0.488 × 0.625 mm^3^. A total of 1–1.2 mL/kg iopromide, 370 mg iodine/ml; (Ultravist, Bayer Schering Pharma, Berlin, Germany) was intravenously administered using a bolus tracking method, with an injection rate of 5 mL/s. Volume rendering and MIP images were reconstructed to aid evaluation.

### Image evaluation

MIP images and source images of CS TOF-MRA and CTA were both used for imaging evaluation. Firstly, CS TOF-MRA images were assessed independently in a randomized order by two neuroradiologists (with 4 and 6 years of experience), who were blinded to any clinical information of the patients. Then, 1 week later, CTA images were presented to the same two neuroradiologists in a randomized order for evaluation. Any disagreement between the 2 readers was resolved by another senior neuroradiologist, who re-evaluated the images and assisted in reaching a consensus agreement (with 10 years of experience).

**Fig. 1:**
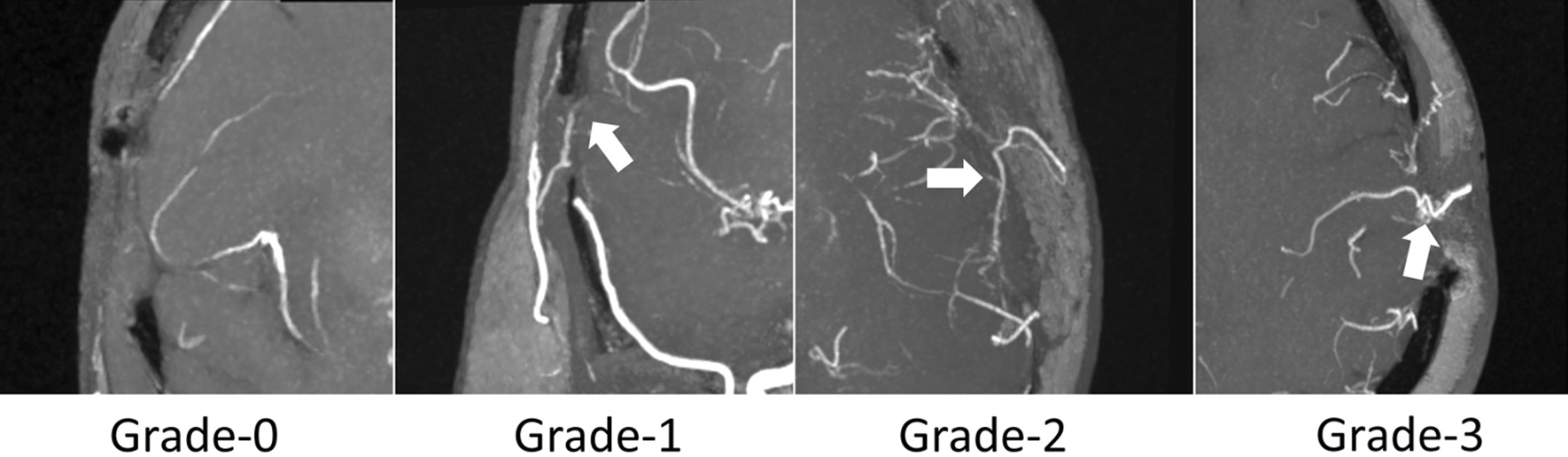
Examples for 4-grades bypasses on CS TOF-MRA. Grade-0, no visualization of bypass; Grade-1, obviously blurred bypass margins with heavily attenuated signal intensity (arrow); Grade-2, slightly blurred vessel margins with slightly attenuated signal intensity; Grade-3, clear vessel margins with strong signal intensity

The diagnostic quality for visualization of STA–MCA bypass was evaluated based on a 4-point scale as followings: Grade-0, no visualization of bypass; Grade-1, obviously blurred bypass margins with heavily attenuated signal intensity/density; Grade-2, slightly blurred vessel margins with slightly attenuated signal intensity/density; Grade-3, clear vessel margins with strong signal intensity/density [20]. Examples for 4-grades bypasses are shown in Fig. 1.

The anastomosis was considered to be patent when the STA was connected with the MCA, and was considered occluded when the STA was not connected with the MCA and MCA branches disappeared on CS TOF-MRA and CTA images [21]. As post-operative DSA could only be obtained from 3 patients in our study, the ground truth for the patency of the anastomosis in most patients was decided by consensus agreement between one senior neuroradiologist and one experienced neurosurgeon (with 21 and 25 years of experience, respectively) after they reviewed all the CS TOF-MRA and CTA images as well as the clinical information, respectively.

**Fig. 2:**
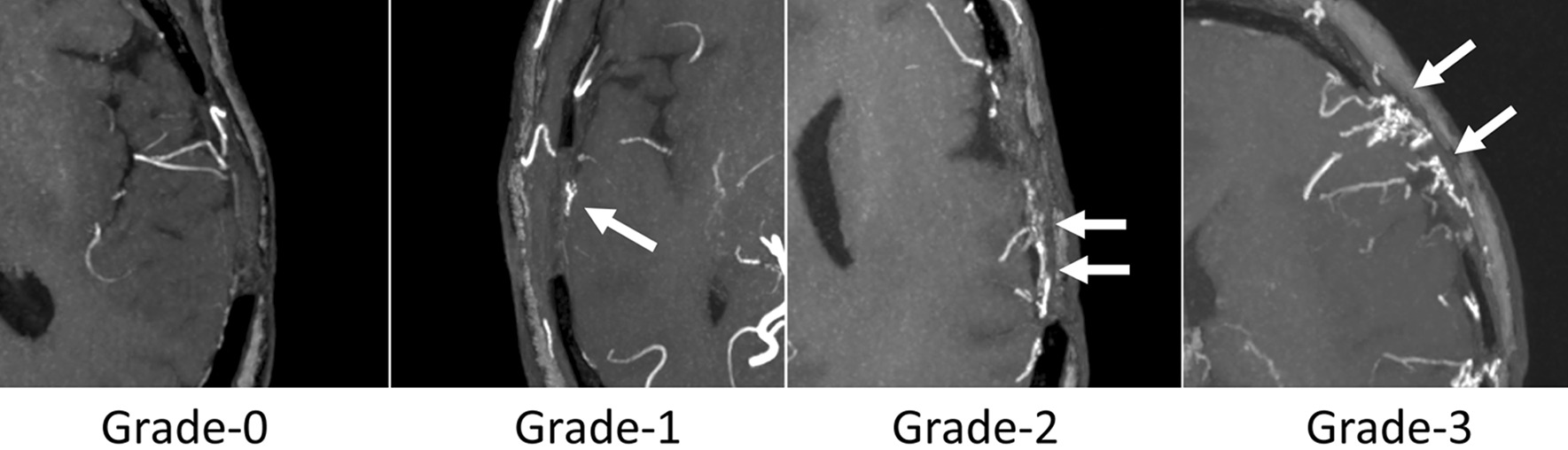
Examples for 4-grades neovascularization on CS TOF-MRA. Grade-0, almost no visualization of neovascularization; Grade-1, a few localized neovascularization, covering less than 25% of the operation area; Grade-2, moderate neovascularization, covering 25–50% of the operation area; Grade-3, abundant neovascularization, covering more than 50% of the operation area

The quality of neovascularization visualization was evaluated on a 4-point scale. Grade-0, almost no visualization of neovascularization; Grade-1, a few localized neovascularization, covering less than 25% of the operation area; Grade-2, moderate neovascularization, covering 25–50% of the operation area; Grade-3, abundant neovascularization, covering more than 50% of the operation area [3, 22]. Examples for 4-grades neovascularization are shown in Fig. 2.

The visibility of MMVs at the basal ganglia and periventricular area was assessed and scored as follows: Grade-0, not visible; Grade-1, scarcely visible (vessel segments were visualized but inadequate for diagnosis); Grade-2, visible (vessel segments are visualized and adequate for diagnosis, but vessel tissue contrast does not appear to be high); Grade-3, excellent (vessel segments were clearly and continuously visualized, and vessel tissue contrast was high) [23]. All criteria for evaluating MMD patients after surgical revascularization are summarized in Table 1.

**Table 1:** Criteria for evaluating MMD patients after surgical revascularization

Criterion and Interpretation	Score
*Visualization of bypass*	
Poor: almost no visualization	0
Moderate: blurred vessel margins with heavily attenuated signal intensity/density	1
Good: blurred vessel margins with slightly attenuated signal intensity/density	2
Excellent: clear vessel margins with intense signal/density	3
*Visualization of anastomosis patency*	
Occluded	0
Patent	1
*Visualization of neovascularization*	
Null: almost no visualization	0
Localized: covering less than 25% of the operation area	1
Moderate: covering 25% to 50% of the operation area	2
Abundant: covering more than 50% of the operation area	3
*Visualization of Moyamoya vessels*	
Not visible	0
Scarcely visible (vessel segments were visualized but inadequate for diagnosis)	1
Visible (vessel segments are visualized and adequate for diagnosis, but vessel-tissue contrast does not appear to be high)	2
Excellent (vessel segments were clearly and continuously visualized, and vessel-tissue contrast was high)	3

### Statistical analysis

Continuous data were summarized as mean ± SD or median (interquartile range presented as the 25th and 75th percentile, IQR). Categoric data were recorded as counts and percentages. The interreader agreement was assessed by the weighted kappa statistic. Kappa value < 0.4 was characterized as poor, those 0.4–0.8 were fair to good, and those > 0.8 were considered excellent. The vessel visualization grades on CS TOF-MRA and CTA were compared using the Wilcoxon signed-rank test or Chi-square test. All data were analyzed statistically using SPSS 22.0 (IBM Corporation; formerly SPSS Inc.). The *P* value was two-sided, and statistical significance was set as *P* < 0.05.

## Results

### Patient characteristic

Twenty consecutive patients met the inclusion criteria were enrolled in this study. Two patients were excluded due to motion artifact which distorted the area of concern. The median age at the time of surgery was 49 years with an age range of 13–60 years. Ten (50.0%) of these patients were male. Preoperatively, three patients were diagnosed with unilateral MMD and 17 were diagnosed with bilateral MMD. Twelve patients presented with ischemic stroke, 1 had transient ischemic attack, 4 had hemorrhagic stroke, and 3 had headaches and dizziness before the operation.

Two patients with bilateral MMD underwent surgical revascularization two times in our hospital. Sixteen hemispheres received combined revascularization (STA–MCA anastomosis combined with EDAMS), and 6 sides had direct revascularization (STA–MCA anastomosis). The time interval between the operation and MR exam was 11 months. The time interval between post-surgical MR exam and CTA was 5 days (Table 2).

**Table 2:** Clinical information of the included subjects

Characteristic	Number
Age (years), median (IQR)	49 (35,53)
Male, n (%)	10 (50.0)
Diagnosis, n (%)	
Bilateral MMD	17 (85.0)
Unilateral MMD	3 (15.0)
Clinical presentation, n (%)	
Ischemic infarction	12 (60.0)
TIA	1 (5.0)
Hemorrhage	4 (20.0)
Headache and dizzy	3 (15.0)
Sides, n (%)	
Right	11 (50.0)
Left	11 (50.0)
Revascularization type (sides), n (%)	
Direct revascularization	6 (27.3)
Combined revascularization	16 (72.7)
Time interval between CS TOF-MRA and operation (months), median (IQR)	11 (4.5,25)
Time interval between CS TOF-MRA and CTA (days), median (IQR)	5 (4,9)

2 patients underwent bilateral bypass; *IQR* interquartile range presented as the 25th and 75th percentile, *MMD* Moyamoya disease, *TIA* transient ischemic attack, *CS TOF-MRA* compressed sensing time-of-flight magnetic resonance angiography, *CTA* computed tomography angiography

### Comparison between CS TOF-MRA and CTA for STA–MCA bypass evaluation

The interobserver agreement in assessing the diagnostic quality of bypass visualization was good to excellent for CS TOF-MRA (*κ* = 0.844) and CTA (*κ* = 0.758), respectively. Thirteen (59.1%) bypasses were ranked as grade 3 on CS TOF-MRA images, in comparison to 5 (22.7%) bypasses on CTA images. The diagnostic grade of bypass visualization on CS TOF-MRA (59.1% grade 3) was significantly higher than that of CTA (22.7% grade 3) (*P* = 0.011, Table 3).

**Table 3:** Comparison between CS TOF-MRA and CTA for evaluating MMD patients after surgical revascularization

	CS TOF-MRA	CTA	*P* value*
STA–MTA bypass visualization, n (%)			0.011
Grade 0	4 (18.2)	5 (22.7)	
Grade 1	1 (4.5)	7 (31.8)	
Grade 2	4 (18.2)	5 (22.7)	
Grade 3	13 (59.1)	5 (22.7)	
Neovascularization, n (%)			0.005
Grade 0	9 (40.9)	15 (68.2)	
Grade 1	7 (31.8)	4 (18.2)	
Grade 2	3 (13.6)	3 (13.6)	
Grade 3	3 (13.6)	0 (0)	
Moyamoya vessels, n (%)			0.036
Grade 0	0 (0.0)	1 (5.0)	
Grade 1	1 (5.0)	6 (30.0)	
Grade 2	10 (50.0)	9 (45.0)	
Grade 3	9 (45.0)	4 (20.0)	

*STA* superficial temporal artery, *MCA* middle cerebral artery, *CS TOF-MRA* compressed sensing time-of-flight magnetic resonance angiography, *CTA* computed tomography angiography

**Fig. 3:**
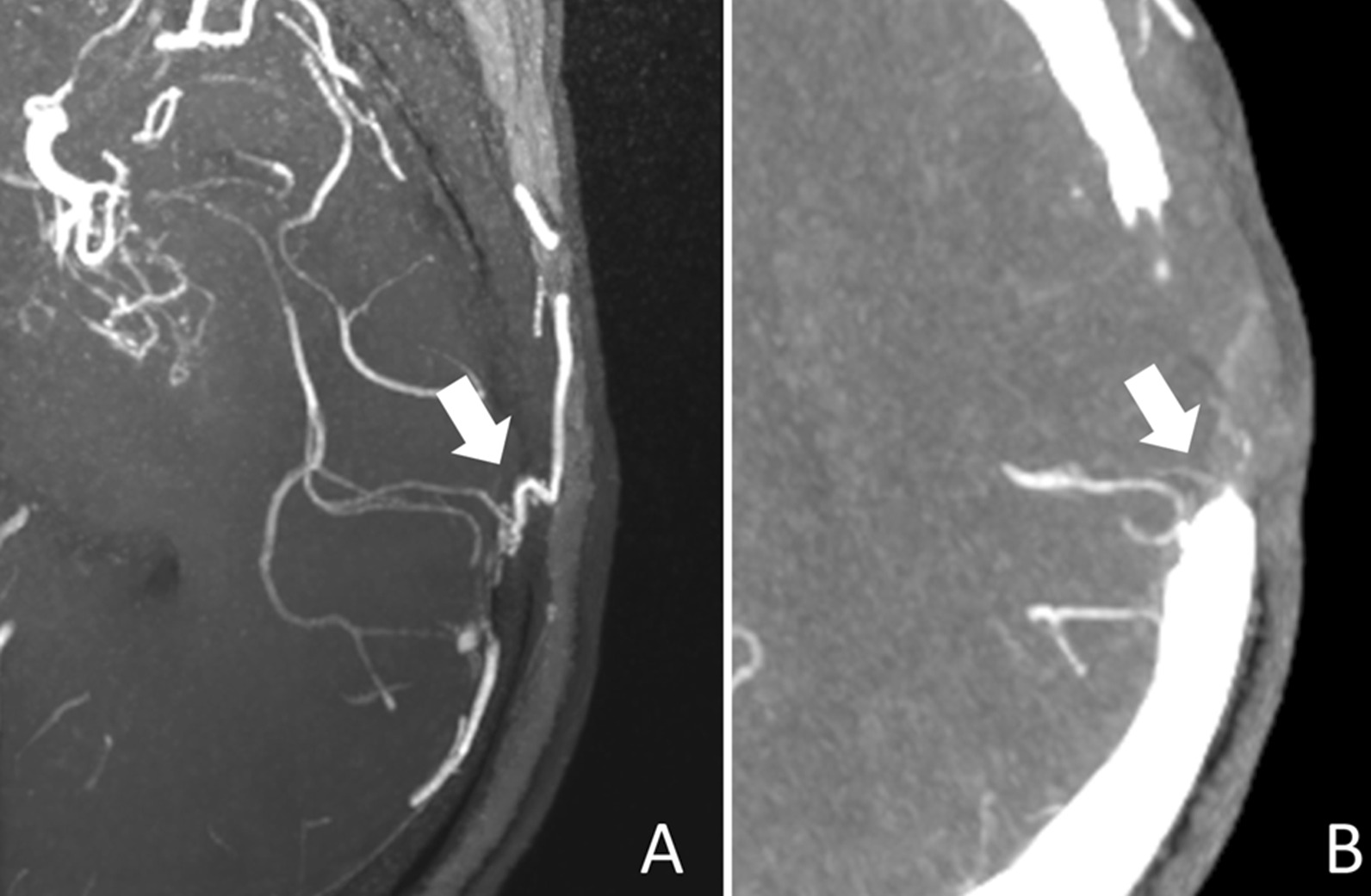
MIP images of a 50-year-old man 17 months after surgical revascularization. The CS TOF-MRA shows the patent anastomosis (**A**), which is considered occluded on CTA (**B**). Note the increased background noise and the interference from the skull on CTA

According to the post-operative DSA (n = 3) and the consensus agreement (n = 19) between two senior doctors, the anastomoses were classified as patent in 19 (86.4%) of 22 bypasses (the ground truth). Eighteen anastomoses were assessed patent on CS TOF-MRA versus 14 on CTA images. Five anastomoses were considered occluded on CTA images, but were definitely patent on CS TOF-MRA (Fig. 3). One anastomosis was considered occluded on CS TOF-MRA but were found patent on CTA. The sensitivity, specificity, positive predictive value, and negative predictive value of CS TOF-MRA were 94.7%, 100%, 100% and 75%, respectively, and were 73.7%, 100%, 100% and 37.5% respectively or CTA (Table 4). The interobserver agreement in assessing the anastomosis patency was excellent for both CS TOF-MRA (*κ* = 1.000) and CTA (*κ* = 0.909).

**Table 4:** Comparison between CS TOF-MRA and CTA for assessing anastomosis patency

	Sensitivity (%)	Specificity (%)	Positive prediction value (%)	Negative predictive value (%)
CS TOF-MRA (%)	94.7	100	100	75
CTA (%)	73.7	100	100	37.5

*CS TOF-MRA* compressed sensing time-of-flight magnetic resonance angiography, *CTA* computed tomography angiography

### Comparison between CS TOF-MRA and CTA for neovascularization visualization

**Fig. 4:**
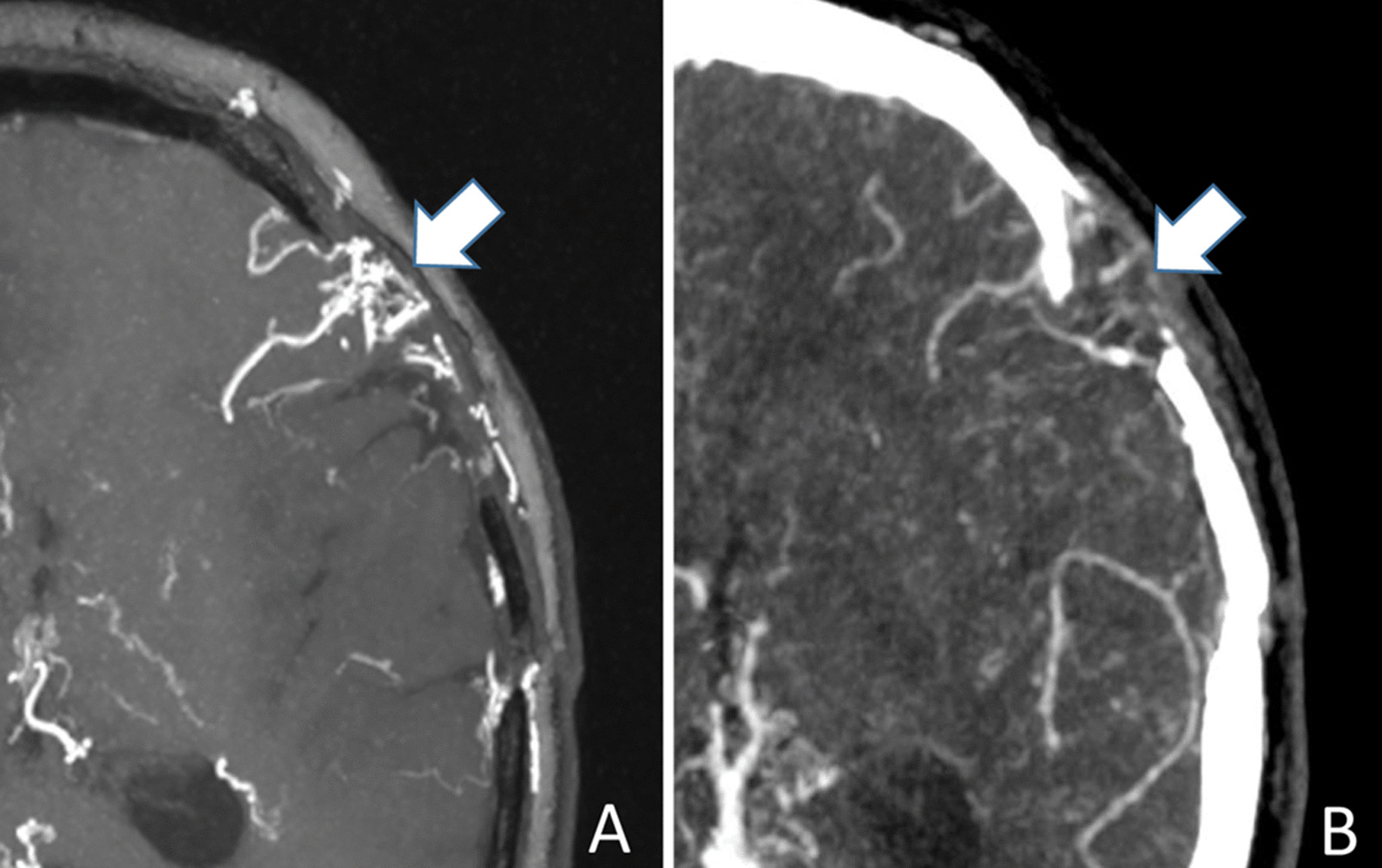
MIP images of a 13-year-old female patient 7 months after surgical revascularization. Collateral formation from neovascularization into the brain pial surface is ranked as Grade-3 on CS TOF-MRA (**A**), while which is ranked as Grade-2 on CTA (**B**)

The interobserver agreement for assessing neovascularization was good for CS TOF-MRA (*κ* = 0.738) and CTA (*κ* = 0.743), respectively. Neovascularization was observed in 13 (59.1%) sides based on CS TOF-MRA images, in comparison to 7 (31.8%) sides on CTA images (Table 3). Neovascularization of 6 sides was verified on CS TOF-MRA, yet could not be found on CTA, due to the interference from the skull and background noise. Neovascularization of 5 sides was ranked as grade 1 or 2 on CTA, but increased to 2 or 3 on CS TOF-MRA (Fig. 4). Grade 3 (abundant neovascularization covering more than 50% of the operation area) was only observed on CS TOF-MRA. Neovascularization was better visualized on CS TOF-MRA compared with CTA (*P* = 0.005).

### Comparison between CS TOF-MRA and CTA for MMVs visualization

**Fig. 5:**
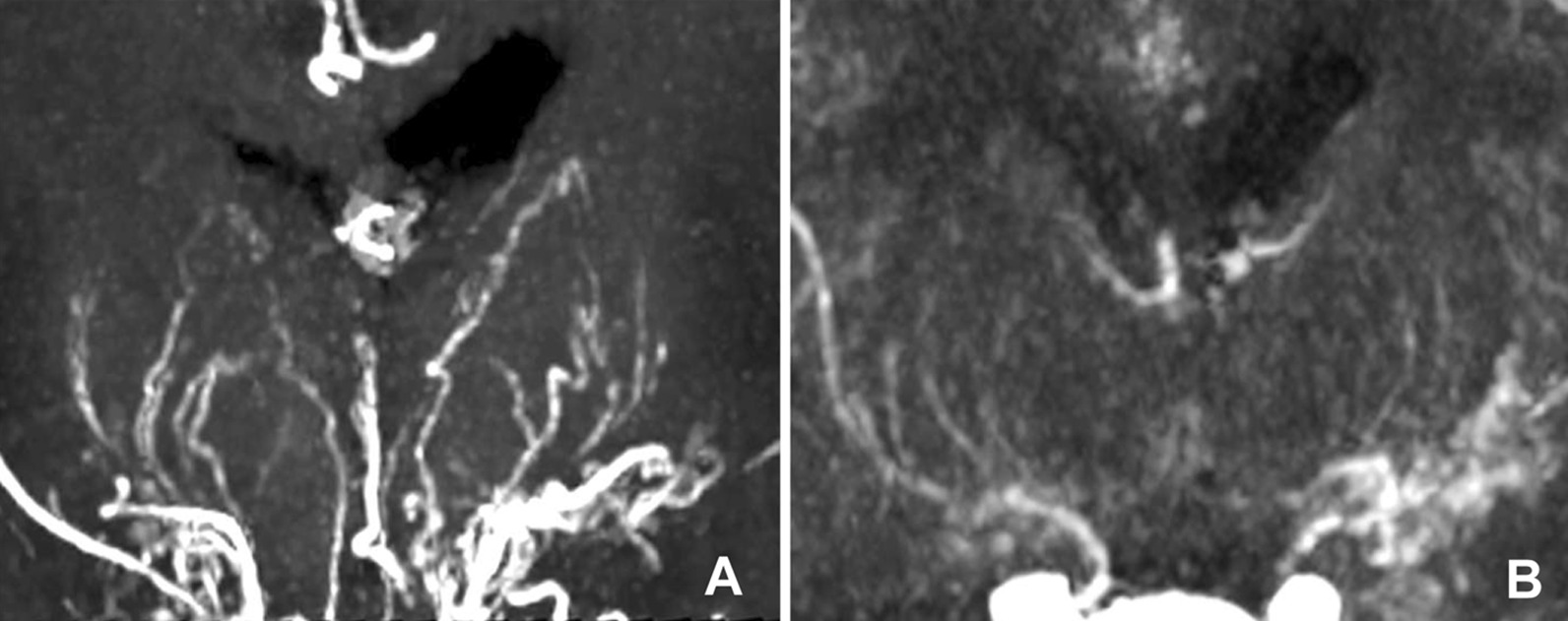
MIP images of a 13-year-old female patient 7 months after surgical revascularization. Moyamoya vessels are clearly and continuously visualized with high vessel-tissue contrast on CS TOF-MRA (Grade-3, **A**), but are inadequate for diagnosis on CTA (Grade-1, **B**)

The interobserver agreement for assessing MMVs was excellent for CS TOF-MRA (*κ* = 0.909) and CTA (*κ* = 0.851), respectively. The visualization of MMVs were ranked as grade 2 and 3 in 19 of 20 (95.0%) MMD patients on CS TOF-MRA images, in comparison to 13 (65.0%) patients on CTA images. The diagnostic grade of MMVs visualization on CS TOF-MRA was significantly higher than that of CTA (*P* = 0.036, Table 3, Fig. 5).

## Discussion

In this study we evaluated the utility of high-resolution (0.4 × 0.4 × 0.4 mm^3^, isotropic) CS TOF-MRA for assessment of MMD patients after surgical revascularization. We found that high-resolution CS TOF-MRA could provide better visualization for STA–MCA bypass, neovascularization and MMVs than traditional CTA within a clinically reasonable time.

The Cho et al. study demonstrated that combined revascularization surgery could achieve favorable long-term outcome [24]. However, given the risk of recurrent infarction or hemorrhage, long-term follow-up and imaging monitoring should be continued after revascularization. The primary advantages of CS TOF-MRA, such as noninvasive imaging without radiation exposure, no additional contrast agent required and imaging easy to be combined with other routine MR sequences (for example, diffusion-weighted imaging or arterial-spin-labeling imaging), make it to be an ideal imaging modality for assessing the angiographic outcome after surgical revascularization [25].

In this current study, the scan time of CS TOF-MRA for achieving a high acquired resolution (0.6 × 0.6 × 0.6 mm^3^) was 5 min and 4 s. By using the same resolution and coverage to CS TOF-MRA, a conventional TOF-MRA with parallel imaging (GRAPPA 2, phase and slice partial Fourier, 7/8) would take 10 min and 8 s. Therefore, the acquisition time was significantly reduced. An acceptable scan duration is beneficial for MMD patients because of the repeated MR examinations. Moreover, a reasonable scan time can expect less motion artifacts and benefit for adding into the routine clinical MR protocols.

Our previous study showed that CS TOF-MRA could provide higher edge sharpness of the intracranial arteries than conventional TOF-MRA [16]. Yamamoto et al. compared CS TOF-MRA with different acceleration factors (CS 3 versus CS 5) for evaluating collaterals in MMD patients. They found that CS with lower acceleration factor (CS 3) better visualized Moyamoya collaterals [19].Another studies of Fushimi et al. and Ding et al. also supported that small arteries (anterior choroidal arteries, distal branches like A2/3, M4, P4 segments) could be better visualized by CS TOF-MRA with an acceleration factor less than 6 [18, 26]. Therefore, considering that the bypass and collateral vessels are all with small size, we set the acceleration factor as 5. Our results indicated that CS TOF-MRA with an acceleration factor of 5 could provide sufficient diagnostic quality and more clear visualization of STA–MCA bypass, neovascularization and MMVs than traditional CTA while keeping a clinically reasonable scan time (5 min).

Several advantages make CS TOF-MRA possible to better depict the bypass, neovascularization and MMVs than traditional CTA. First, TOF-MRA is based on the principle of flow-related enhancement, which highlights the vessel signal and suppresses the background signal. By combining TOF-MRA with CS, the image quality of CS TOF-MRA gets better by enhancing the contrast of vessels due to inherently denoising procedure of CS reconstruction [19]. Second, the spatial resolution of CS TOF-MRA was very high and isotropic (0.4 × 0.4 × 0.4 mm^3^), which was benefit for image reconstruction and better display the small vessels. In contrast, CTA has several limitations. When the bypass enhances together with the brain parenchyma on CTA, increased signals from background noise will interfere with the evaluation of the bypass vessels. And the trepanation segments of the bypass on CTA are usually difficult to be observed because of the interference from the skull.

There were several limitations to our study. First, only 3 patients had DSA as a gold standard. The reason for this is that DSA is not considered essential in clinical practice for following up of MMD patients after surgical revascularization. Eighteen of 20 patients in our study had no clinical symptoms at the time of follow-up. Therefore, these patients underwent noninvasive examinations instead of DSA. Second, this is a single-center study involving a relatively small number of MMD patients. Larger cohorts are needed for validation before applying CS TOF-MRA to routine postoperative follow-up workflow. Third, the number of iterations was fixed at 10 in the current study, consistent with a previous study from Yamamoto et al. [18]. Vessel sharpness and the visibility of small vessels may be improved with more iterations [27]. However, the reconstruction time would be largely increased when using an iteration number of 20, which may affect the clinical workflow.

In conclusion, CS TOF-MRA performed better than CTA for assessing STA–MCA bypass, neovascularization and MMVs in MMD patients after surgical revascularization in a reasonable scan time. Considering its advantages (noninvasive imaging without radiation and no contrast medium required) which can be easily combined with other MR techniques, CS TOF-MRA may be beneficial to improve the imaging workflow for MMD patients. In particular, pediatric patients and those who need to receive lifelong follow-up examinations repeatedly could benefit from this novel approach.

## Data Availability

The data and materials are available from the corresponding author on reasonable request.

## Abbreviations

CS TOF-MRA: Compressed sensing time-of-flight magnetic resonance angiography
EDAMS: Encephalo-duroarterio-myo-synangiosis
MMD: Moyamoya disease
MCA: Middle cerebral artery
STA: Superficial temporal artery
MMVs: Moyamoya vessels

## References

[1] Guidelines for diagnosis and treatment of Moyamoya disease (spontaneous occlusion of the circle of willis). Neurol Med Chir (Tokyo) 2012;52(5):245–66. 10.2176/nmc.52.245 22870528

[2] Acker G, Fekonja L, Vajkoczy P (2018). Surgical management of Moyamoya disease. Stroke.

[3] Imai H (2015). The importance of encephalo-myo-synangiosis in surgical revascularization strategies for Moyamoya disease in children and adults. World Neurosurg.

[4] Ge P (2019). Angiographic outcomes of direct and combined bypass surgery in Moyamoya disease. Front Neurol.

[5] Yoon S, Burkhardt JK, Lawton MT (2018). Long-term patency in cerebral revascularization surgery: an analysis of a consecutive series of 430 bypasses. J Neurosurg.

[6] Lucia K (2021). Surgical management of failed revascularization in Moyamoya vasculopathy. Front Neurol.

[7] Zhai X (2018). Risk factors associated with neurologic deterioration after combined direct and indirect revascularization in patients with Moyamoya disease on the east coast of china. World Neurosurg.

[8] Ge P (2020). Postoperative collateral formation after indirect bypass for hemorrhagic Moyamoya disease. BMC Neurol.

[9] Thines L (2009). Assessment of extracranial–intracranial bypass patency with 64-slice multidetector computerized tomography angiography. Neuroradiology.

[10] Hurth H (2021). Early post-operative CT-angiography imaging after EC-IC bypass surgery in Moyamoya patients. Front Neurol.

[11] Tsuchiya K (2013). Postoperative evaluation of superficial temporal artery-middle cerebral artery bypass using an MR angiography technique with combined white-blood and black-blood sequences. J Magn Reson Imaging.

[12] Chen Q (2014). Assessment of extracranial–intracranial bypass in Moyamoya disease using 3T time-of-flight MR angiography: comparison with CT angiography. Vasa.

[13] Feng L (2017). Compressed sensing for body MRI. J Magn Reson Imaging.

[14] Hollingsworth KG (2015). Reducing acquisition time in clinical MRI by data undersampling and compressed sensing reconstruction. Phys Med Biol.

[15] Zhang X (2020). Highly accelerated compressed sensing time-of-flight magnetic resonance angiography may be reliable for diagnosing head and neck arterial steno-occlusive disease: a comparative study with digital subtraction angiography. Eur Radiol.

[16] Lu SS (2018). Clinical evaluation of highly accelerated compressed sensing time-of-flight MR angiography for intracranial arterial stenosis. AJNR Am J Neuroradiol.

[17] Lin Z (2019). Clinical feasibility study of 3D intracranial magnetic resonance angiography using compressed sensing. J Magn Reson Imaging.

[18] Yamamoto T (2016). Time-of-flight magnetic resonance angiography with sparse undersampling and iterative reconstruction: comparison with conventional parallel imaging for accelerated imaging. Investig Radiol.

[19] Yamamoto T (2018). Magnetic resonance angiography with compressed sensing: an evaluation of Moyamoya disease. PLoS ONE.

[20] Tang H (2019). Accelerated time-of-flight magnetic resonance angiography with sparse undersampling and iterative reconstruction for the evaluation of intracranial arteries. Korean J Radiol.

[21] Wang M (2021). Vessel-selective 4D MRA based on ASL might potentially show better performance than 3D TOF MRA for treatment evaluation in patients with intra-extracranial bypass surgery: a prospective study. Eur Radiol.

[22] Zhao Y (2019). Predictors of neoangiogenesis after indirect revascularization in Moyamoya disease: a multicenter retrospective study. J Neurosurg.

[23] Fushimi Y (2006). Comparison of 3.0- and 1.5-T three-dimensional time-of-flight MR angiography in Moyamoya disease: preliminary experience. Radiology.

[24] Cho WS (2014). Long-term outcomes after combined revascularization surgery in adult Moyamoya disease. Stroke.

[25] Sekine T (2016). 4D flow MRI assessment of extracranial–intracranial bypass: qualitative and quantitative evaluation of the hemodynamics. Neuroradiology.

[26] Ding J (2021). Acceleration of brain TOF-MRA with compressed sensitivity encoding: a multicenter clinical study. AJNR Am J Neuroradiol.

[27] Stalder AF (2015). Highly undersampled contrast-enhanced MRA with iterative reconstruction: integration in a clinical setting. Magn Reson Med.

